# SARS-CoV-2 Omicron Variant in Patients With Chronic Lymphocytic Leukemia: Case Series

**DOI:** 10.7759/cureus.32041

**Published:** 2022-11-30

**Authors:** Ibrahim Khamees, Elrazi A Ali, Lujain Malkawi, Waail Rozi, Mohamed A Yassin

**Affiliations:** 1 Internal Medicine, Hamad Medical Corporation, Doha, QAT; 2 Internal Medicine, Interfaith Medical Center/One Brooklyn Health, Brooklyn, USA; 3 Internal Medicine, Jordan University of Science and Technology, Irbid, JOR; 4 Hematology and Oncology, Hamad General Hospital, Doha, QAT

**Keywords:** sars-cov-2 vaccine, sars-cov-2, omicron, covid-19, chronic lymphocytic leukemia (cll)

## Abstract

The Coronavirus disease 2019 (COVID-19) is considered the largest pandemic in modern history. Since the first case was reported in 2019, several mutations have affected the severe acute respiratory syndrome-Coronavirus-2 (SARS-CoV-2), resulting in the emergence of new strains. These strains vary significantly in severity and transmissibility. The Omicron (B.1.1.529) variant was reported to cause mild disease in those affected, but little is known about the effect of Omicron on patients with chronic lymphocytic leukemia (CLL). We are reporting a case series of three patients with CLL who experienced infection with the SARS-CoV-2 Omicron variant and their outcomes.

## Introduction

COVID-19 was declared a pandemic on March 11, 2020, by the World Health Organization (WHO). The clinical course of COVID-19 infection varies from completely asymptomatic infection to severe acute respiratory distress syndrome [1]. Patients with specific risk factors are considered at greater risk of having worse symptoms and a worse prognosis due to COVID-19 infection than others. These risk factors include obesity, old age, hypertension, diabetes mellitus, and other medical comorbidities [2,3]. Patients with cancer, including hematologic malignancies, were reported to have a worse COVID-19 infection compared to those without cancer [4]. Multiple SARS-CoV-2 variants have been discovered since the start of the pandemic; the most recent variant is the Omicron, which was first reported in November 2021 [5]. Chronic lymphocytic leukemia (CLL) is a malignancy that mostly affects those over 50. It is characterized by the proliferation of one clone of neoplastic B lymphocytes in the lymph nodes [6]. Patients with CLL have high mortality and morbidity after COVID-19 infection [7]. Only a few unpublished papers discussed the risk of the Omicron variant in patients with CLL. We aim to shed some light on the impact of the Omicron variant of COVID-19 on patients with CLL by summarizing the clinical course and the outcomes of 3 patients with CLL who were infected with this variant.

## Case presentation

Case 1

**Figure 1:**
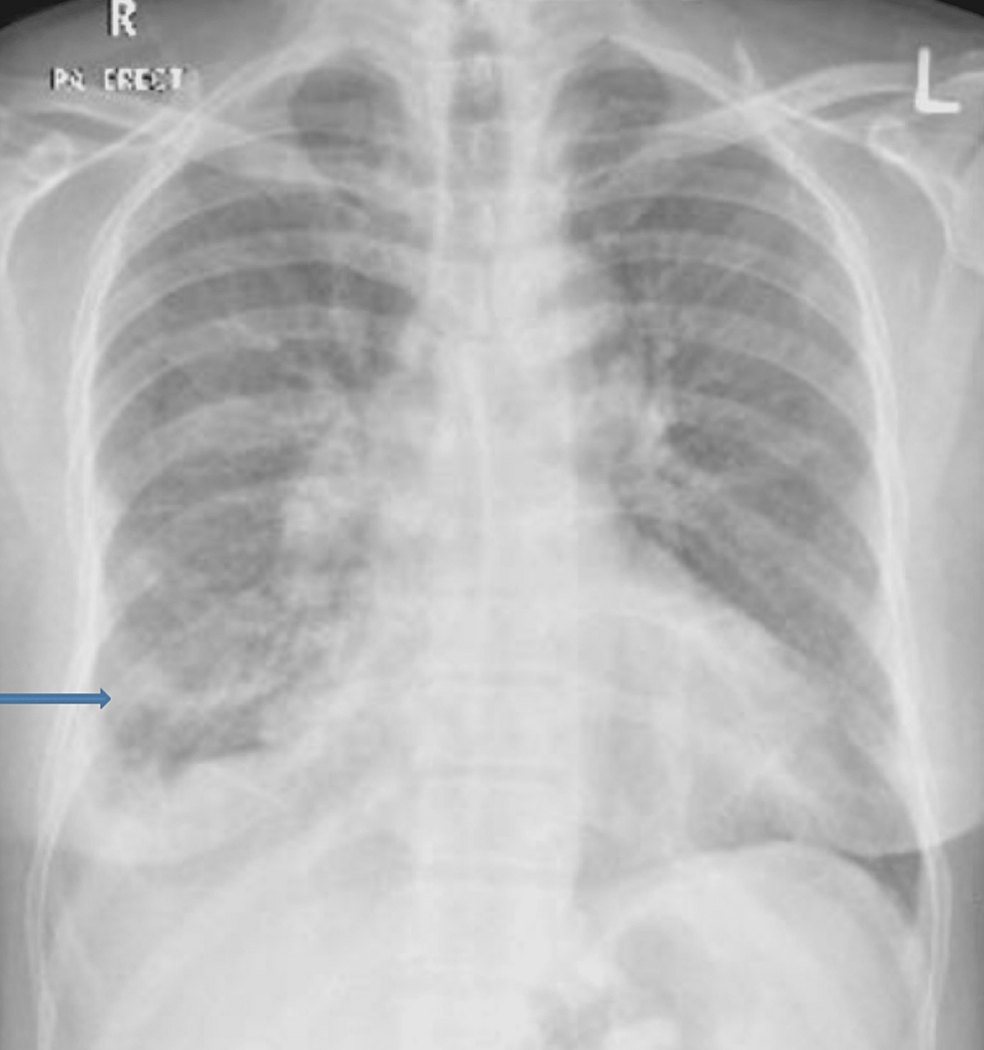
Chest X-ray Patchy infiltrates and hazy opacities in both lungs predominantly in the right lower zone with right-sided pleural effusion (arrows).

A 63-year-old male was previously known to have hypertension, diabetes mellitus, hypothyroidism, and CLL, which was diagnosed six years ago and for which he was receiving ibrutinib 420 mg daily. The patient had not received the COVID-19 vaccine before. He presented with a fever, cough, and shortness of breath. His CRP was 56 mg/L (normal range: <5). His chest X-ray was significant for patchy infiltrates and hazy opacities in both lungs, predominantly in the right lower zone with right-sided pleural effusion (Figure 1). His COVID-19 antigen test came back positive during the Omicron wave, and he was admitted for treatment of COVID-19 pneumonia. He required 4 liters of oxygen on admission. He received remdesivir, dexamethasone, and piperacillin/tazobactam for five days, resulting in rapid improvement and discharge from the hospital.

Case 2

An 80-year-old Arabic male patient was previously known to have hypertension, diabetes mellitus, chronic kidney disease stage 3, asthma, coronary artery disease, and CLL, which had been diagnosed six years earlier and for which he was receiving ibrutinib 420 mg daily. He received three doses of the Pfizer COVID-19 vaccine, and the last dose was received 109 days before this presentation. He presented with a fever and cough for two days. His CRP was 83 mg/L (normal range: <5), and his chest X-ray was normal, but his COVID-19 antigen test came back positive during the Omicron wave. He was admitted for treatment of a COVID-19 upper respiratory tract infection. He received one dose of Sotrovimab 500 mg infusion and was discharged one day later without symptoms.

Case 3

**Figure 2:**
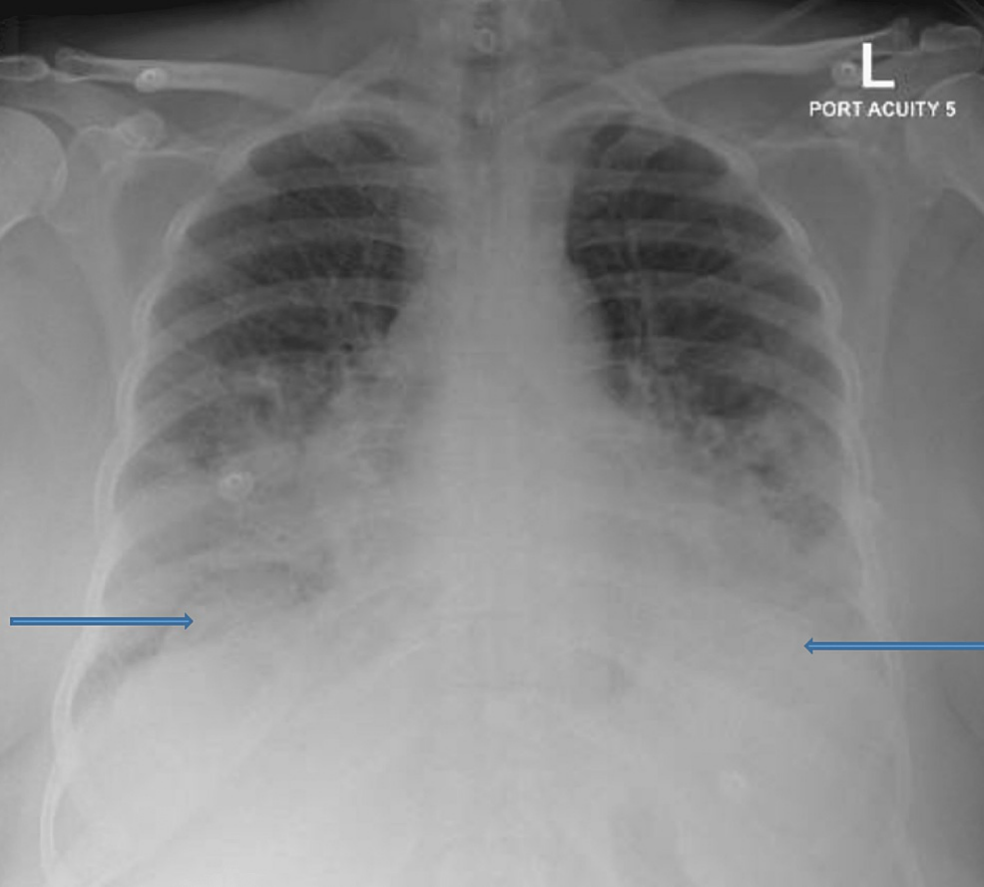
Chest X-ray Bilateral prominent broncho-vascular markings with lower lobe consolidation, in addition to bilateral pleural effusion (arrows).

A 58-year-old female patient, previously known to have asthma, diabetes mellitus, and CLL, was diagnosed one year before this presentation. She had received six cycles of chemotherapy previously in the form of bendamustine and rituximab, and the last cycle was five months before this presentation. She received two doses of the Pfizer COVID-19 vaccine, the last of which she received three months before this presentation. She presented with a fever and cough for five days. Her oxygen saturation at admission was 95% on 6-liter oxygen. Her labs were significant for the low absolute neutrophil count: (0.6 * 10^3^/µL) and high inflammatory markers. Her chest X-ray showed bilateral prominent broncho-vascular markings with lower lobe consolidation, in addition to bilateral pleural effusion (Figure 2). The COVID-19 PCR test came back positive during the Omicron wave. She was admitted for treatment of COVID-19 pneumonia. She received five days of favipiravir and remdesivir, along with dexamethasone, azithromycin, and ceftriaxone. The hematology team was consulted regarding her neutropenia and low IgG level: 1.84 g/L (normal range: 7-16 g/L), and they advised starting the patient on IVIG at 0.4 mg/kg as the patient is immunocompromised. The patient improved significantly after these measures and was discharged after eight days.

## Discussion

SARS-CoV-2 belongs to the Coronavirus family, which are enveloped, positive single-stranded large RNA viruses that infect humans and are known for their ability to infect various types of animals. The COVID-19 infection mainly presents with respiratory symptoms, inducing a flu-like illness with fever, cough, and shortness of breath. Since its discovery, multiple variants have emerged that are variable in severity. The last variant discovered in November 2021 was named the Omicron variant, which has an infectivity 10 times greater than the original virus and two times greater than the Delta variant, resulting in its rapid spread in most parts of the world [5]. This variant was reported to cause milder disease compared to other variants, and several studies showed that it was associated with a significantly lower case fatality ratio (CFR) and risk of hospitalization when compared to the Delta variant of the SARS-CoV-2 [8].

CLL is the most common adult leukemia in the world, with a median age at diagnosis of 71 years. The usual presentation of CLL is incidental lymphocytosis in the blood count without significant symptoms, but it can also present with lymphadenopathy, recurrent infections, anemia, and other signs of bone marrow failure [9]. CLL, like all hematological malignancies, causes immunosuppression because of the clonal proliferation of lymphocytes, which makes the patients more vulnerable to several infections, including SARS-Cov-2 [10,11]. Many studies were conducted before the Omicron variant's appearance to study the impact of SARS-CoV-2 infection on patients with CLL. A previous study reported that the CFR for the CLL patients infected with SARS-CoV-2 was 27.4% and increased to 38.4% in severe COVID-19 infection cases [12], which was higher when compared to the general population's 17.1% [13]. The same study reported that the hospitalization percentage in these patients was 74.7%, and 25.5% required ICU admission. They also reported that receiving CLL-targeted treatment before or at the time of the infection increased the risk of death from severe COVID-19 infection in patients with CLL [12]. Another study reported a CFR of 28%, a hospital admission percentage of 75%, and an ICU admission rate of 27% in patients with CLL infected with SARS-CoV-2 [6].

After the emergence of the Omicron variant, one study conducted in Denmark [14] compared the impact of this variant to the other variants in patients with CLL; however, it has not been peer-reviewed yet. It was found that hospitalization and admission rates due to COVID-19 infection declined significantly during the Omicron periods in patients with CLL, which could be attributed to the higher vaccination and treatment rates, along with the milder nature of the Omicron variant. However, the mortality rate was 23% during the Omicron dominance period, which is comparable to the other variants [14]. In our report, the three patients were admitted to the hospital but did not require ICU admissions. Two of them required oxygen initially, but all were discharged without significant complications.

Several vaccines were developed to fight the COVID-19 pandemic, and variable protection abilities were noticed against the Omicron variant. Patients with CLL were found to have impaired cellular and humoral immunogenicity following COVID-19 vaccination when compared to controls [15]. The second patient in our report was the only one to receive three doses of the COVID-19 vaccine, and he had the mildest course among them. Patients with chronic myeloid leukemia (CML) and Philadelphia negative myeloproliferative neoplasms were reported to have a better prognosis after Omicron infection compared to patients with CLL [16,17]. In a recent study concerning the impact of the Omicron variant on CML patients, only three patients out of 11 were hospitalized, and no mortality was reported [16]. This might be attributed to the younger age and fewer comorbidities of these patients.

## Conclusions

The Omicron variant is known to cause a mild infection compared to other variants of SARS-CoV-2. But this might not apply to immunocompromised patients like those with CLL. Larger studies are needed to establish the prognosis and the best treatment options for patients with CLL infected with the Omicron variant.
